# Chromomycose cutanée à Fonsecaea Pedrosoi: à propos d’un cas

**DOI:** 10.11604/pamj.2018.30.187.5326

**Published:** 2018-06-28

**Authors:** Kenza Baline, Fouzia Hali

**Affiliations:** 1 Service de Dermatologie et de Vénéréologie, CHU Ibn Rochd, Casablanca, Maroc

**Keywords:** Chromomycose, Fonsecaea Pedrosoi, corps fumagoides, Chromoblastomycosis, Fonsecaea pedrosoi, fumagoid body

## Abstract

We report the case of a 13-year old female patient from rural areas presenting with papulonodular lesion of 4/3cm in the lower third of the right leg, evolving over the last two years. Skin biopsy showed papillomatous hyperplasia with neutrophilic microabscess and spores. Direct mycological examination showed fumagoid bodies and Fonsecaea pedrosoi was isolated from culture. The patient underwent medico-surgical treatment (terbinafine 250mg/day for 6 months + resection and then skin graft) with good evolution and a follow-up period of 2 years without recurrences. Chromoblastomycosis is a chronic fungal skin infection, common in tropical and subtropical areas and rare in North Africa. In Morocco, only seven cases have been reported in the literature until June 2014. It is contracted from inoculation of germ after contact with the soil or organic matters. Responsible officers are pigmented fungi belonging to the dematiated group. Given its rarity, it can mimic other dermatoses such as leishmaniasis or tuberculosis. Despite the rarity of this infection, clinicians should suspect it in patients with chronic skin lesions (verrucous, vegetative, nodular and grouped in patches), especially if they occur in areas exposed to potential plant injuries, such as the lower limbs. Mycological examination is necessary to confirm the diagnosis. Surgery or the association between surgery and systemic antifungal drugs are the treatments of choice because antifungal drugs alone may result in resistances or recurrences.

## Image en médecine

Nous rapportons le cas d’une patiente de 13 ans d’origine rurale qui présentait une lésion papulo-nodulaire de 4/3cm du tiers inférieur de la jambe droite, évoluant depuis deux années. La biopsie cutanée montrait une hyperplasie papillomateuse avec des micro-abcès à polynucléaires neutrophiles et des spores. L’étude mycologique objectivait des corps fumagoides à l’examen direct et isolait Fonsecaea Pedrosoi à la culture. La patiente a bénéficié d’un traitement médico-chirurgical (terbinafine 250mg/j pendant 6 mois + exérèse puis greffe cutanée) avec une bonne évolution, le recul actuel étant de deux années sans récidive. La chromomycose est une infection fongique chronique de la peau, fréquente en zones tropicales et subtropicales et rare au nord de l’Afrique. Au Maroc, jusqu'à juin 2014, sept cas seulement ont été rapportés dans la littérature. Elle est contractée par inoculation du germe après contact avec le sol ou les matières organiques. Les agents responsables sont des champignons pigmentés du groupe des dématiés. Compte tenu de sa rareté, elle peut mimer d’autres dermatoses comme la leishmaniose ou la tuberculose. Malgré la rareté de cette infection, il faut y penser devant des lésions cutanées chroniques (verruqueuses, végétantes, nodulaires, en plaque) surtout si elles sont localisées au niveau des zones exposées à d’éventuels traumatismes végétaux comme les membres inférieurs. Ceci dit, la place de l’étude mycologique parait indéniable pour confirmer le diagnostic. Le traitement de choix est la chirurgie ou l’association chirurgie et antifongiques systémiques car l’utilisation des antifongiques seuls peut se solder par des résistances ou des récidives.

**Figure 1:**
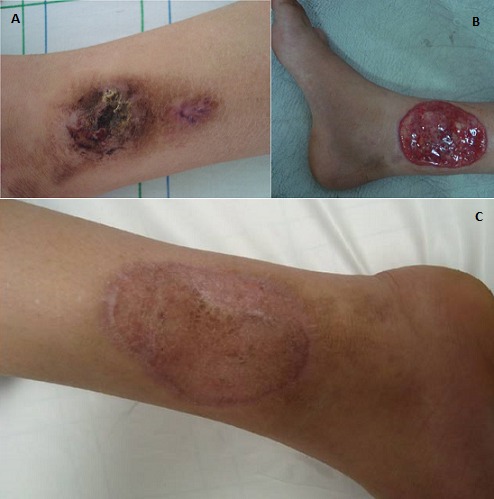
A) lésion papulo-nodulaire du tiers inférieur de la jambe; B) image illustrant la lésion après exérèse chirurgicale; C) image illustrant la lésion après greffe cutanée

